# Pregnancy-related mortality in Africa and Asia: evidence from INDEPTH Health and Demographic Surveillance System sites

**DOI:** 10.3402/gha.v7.25368

**Published:** 2014-10-29

**Authors:** P. Kim Streatfield, Nurul Alam, Yacouba Compaoré, Clementine Rossier, Abdramane B. Soura, Bassirou Bonfoh, Fabienne Jaeger, Eliezer K. Ngoran, Juerg Utzinger, Pierre Gomez, Momodou Jasseh, Akosua Ansah, Cornelius Debpuur, Abraham Oduro, John Williams, Sheila Addei, Margaret Gyapong, Vida A. Kukula, Evasius Bauni, George Mochamah, Carolyne Ndila, Thomas N. Williams, Meghna Desai, Hellen Moige, Frank O. Odhiambo, Sheila Ogwang, Donatien Beguy, Alex Ezeh, Samuel Oti, Menard Chihana, Amelia Crampin, Alison Price, Valérie Delaunay, Aldiouma Diallo, Laetitia Douillot, Cheikh Sokhna, Mark A. Collinson, Kathleen Kahn, Stephen M. Tollman, Kobus Herbst, Joël Mossong, Jacques B.O. Emina, Osman A. Sankoh, Peter Byass

**Affiliations:** 1 Matlab HDSS, Bangladesh; 2 International Centre for Diarrhoeal Disease Research, Bangladesh; 3 INDEPTH Network, Accra, Ghana; 4 AMK HDSS, Bangladesh; 5 Centre for Population, Urbanisation and Climate Change, International Centre for Diarrhoeal Disease Research, Bangladesh; 6 Ouagadougou HDSS, Burkina Faso; 7 Institut Supérieur des Sciences de la Population, Université de Ouagadougou, Burkina Faso; 8 Institut d’Études Démographique et du parcours de vie, Université de Genève, Geneva, Switzerland; 9 Taabo HDSS, Côte d'Ivoire; 10 Centre Suisse de Recherches Scientifiques en Côte d'Ivoire, Abidjan, Côte d'Ivoire; 11 Swiss Tropical and Public Health Institute, Basel, Switzerland; 12 Université Félix Houphoët-Boigny, Abidjan, Côte d'Ivoire; 13 Farafenni HDSS, The Gambia; 14 Medical Research Council, The Gambia Unit, Fajara, The Gambia; 15 Navrongo HDSS, Ghana; 16 Navrongo Health Research Centre, Navrongo, Ghana; 17 Dodowa HDSS, Ghana; 18 Dodowa Health Research Centre, Dodowa, Ghana; 19 School of Public Health, University of Ghana, Legon, Ghana; 20 Kilifi HDSS, Kenya; 21 KEMRI-Wellcome Trust Research Programme, Kilifi, Kenya; 22 Department of Medicine, Imperial College, St. Mary's Hospital, London, United Kingdom; 23 Kisumu HDSS, Kenya; 24 KEMRI/CDC Research and Public Health Collaboration and KEMRI Center for Global Health Research, Kisumu, Kenya; 25 Nairobi HDSS, Kenya; 26 African Population and Health Research Center, Nairobi, Kenya; 27 Karonga HDSS, Malawi; 28 Karonga Prevention Study, Chilumba, Malawi; 29 London School of Hygiene and Tropical Medicine, London, United Kingdom; 30 Niakhar HDSS, Senegal; 31 Institut de Recherche pour le Developpement (IRD), Dakar, Sénégal; 32 Agincourt HDSS, South Africa; 33 MRC/Wits Rural Public Health and Health Transitions Research Unit (Agincourt), School of Public Health, Faculty of Health Sciences, University of the Witwatersrand, Johannesburg, South Africa; 34 Umeå Centre for Global Health Research, Umeå University, Umeå, Sweden; 35 Africa Centre HDSS, South Africa; 36 Africa Centre for Health and Population Studies, University of KwaZulu-Natal, Somkhele, KwaZulu-Natal, South Africa; 37 National Health Laboratory, Surveillance & Epidemiology of Infectious Diseases, Dudelange, Luxembourg; 38 School of Public Health, Faculty of Health Sciences, University of the Witwatersrand, Johannesburg, South Africa; 39 Hanoi Medical University, Hanoi, Vietnam; 40 WHO Collaborating Centre for Verbal Autopsy, Umeå Centre for Global Health Research, Umeå University, Umeå, Sweden

**Keywords:** maternal mortality, cause of death, Africa, Asia, verbal autopsy, INDEPTH Network

## Abstract

**Background:**

Women continue to die in unacceptably large numbers around the world as a result of pregnancy, particularly in sub-Saharan Africa and Asia. Part of the problem is a lack of accurate, population-based information characterising the issues and informing solutions. Population surveillance sites, such as those operated within the INDEPTH Network, have the potential to contribute to bridging the information gaps.

**Objective:**

To describe patterns of pregnancy-related mortality at INDEPTH Network Health and Demographic Surveillance System sites in sub-Saharan Africa and southeast Asia in terms of maternal mortality ratio (MMR) and cause-specific mortality rates.

**Design:**

Data on individual deaths among women of reproductive age (WRA) (15–49) resident in INDEPTH sites were collated into a standardised database using the INDEPTH 2013 population standard, the WHO 2012 verbal autopsy (VA) standard, and the InterVA model for assigning cause of death.

**Results:**

These analyses are based on reports from 14 INDEPTH sites, covering 14,198 deaths among WRA over 2,595,605 person-years observed. MMRs varied between 128 and 461 per 100,000 live births, while maternal mortality rates ranged from 0.11 to 0.74 per 1,000 person-years. Detailed rates per cause are tabulated, including analyses of direct maternal, indirect maternal, and incidental pregnancy-related deaths across the 14 sites.

**Conclusions:**

As expected, these findings confirmed unacceptably high continuing levels of maternal mortality. However, they also demonstrate the effectiveness of INDEPTH sites and of the VA methods applied to arrive at measurements of maternal mortality that are essential for planning effective solutions and monitoring programmatic impacts.

The tragedy of women dying in the context of being pregnant or giving birth continues to be a major, but almost entirely avoidable, problem. A number of countries consistently achieve a maternal mortality ratio (MMR) of less than 10 maternal deaths per 100,000 live births, but the global MMR remains at levels of several hundred mothers dying for every 100,000 births (1). Most pregnancy-related deaths (PRD) occur in the world's poorer countries, and, irrespective of medical causes of death, a proportion are due to health systems failures such as ineffective referral and transport systems in cases of emergency. In addition, health information systems are generally weakest where the problem of pregnancy-related mortality is the greatest (2).

Much information on pregnancy-related mortality comes from maternal death surveys of various kinds, including Demographic and Household Surveys (DHS) (3), and often involves indirect methods of identifying maternal deaths, such as the sisterhood method (4). Data from health facilities are also often used, even though 1) many women do not use facilities for their deliveries, and 2) facilities tend to attract complicated cases. Because all these approaches do not directly access the details of deaths as and when they occur in communities, they are subject to a range of biases and uncertainties which have often hindered understanding of pregnancy-related mortality patterns. Depending on methods used, it may also be difficult to separate maternal deaths (direct and indirect maternal causes) from all PRD (any deaths occurring during or within 6 weeks of pregnancy). Global estimates of maternal mortality, made both by the United Nations Maternal Mortality Interagency Estimates Group (MMEIG) (1) and the Institute of Health Metrics and Evaluation (IHME) (5) rely heavily on whatever survey and other data happens to be available at the country level, and consequently are influenced by the same uncertainties.

Because INDEPTH Network Health and Demographic Surveillance sites (HDSS) follow vital events in defined populations on a continuous basis, they are able to document pregnancy-related mortality directly (6). Furthermore, since all deaths among women of reproductive age (WRA) are followed up and subject to verbal autopsy (VA) interviews, which include questions about the woman's pregnancy status irrespective of her cause of death, it is possible to look at pregnancy-related mortality as cause-specific mortality rates among WRA, and to assess the effect of pregnancy as a risk factor for all causes of death. This also enables estimation of maternal deaths as a subset of all pregnancy-related mortality, which, together with demographic information on births, allows calculation of MMRs.

Our aim in this paper is to address these issues on the basis of a dataset collected at 22 INDEPTH Network HDSSs across Africa and Asia. Although these sites are not constituted as a representative sample, they provide point estimates over a wide range of settings and time periods.

## Methods

The overall INDEPTH dataset, available from the INDEPTH Data Repository (7), from which these pregnancy-specific analyses are drawn, is described in detail elsewhere (8). For WRA, the dataset documents 15,295 deaths in 3,098,718 person-years of observation across 22 sites. The methods involved in compiling the overall dataset are summarised in Box 1.

*Box 1*. Summary of methodology based on the detailed description in the introductory paper (8).

**Age–sex–time standardisation**

To avoid effects of differences and changes in age–sex structures of populations, mortality fractions and rates have been adjusted using the INDEPTH 2013 population standard (9). A weighting factor was calculated for each site, age group, sex, and year category in relation to the standard for the corresponding age group and sex, and incorporated into the overall dataset. This is referred to in this paper as age–sex–time standardisation in the contexts where it is used.

**Cause of death assignment**

The InterVA-4 (version 4.02) probabilistic model was used for all the cause of death assignments in the overall dataset (10). InterVA-4 is fully compliant with the WHO 2012 Verbal Autopsy standard and generates causes of death categorised by ICD-10 groups (11). The data reported here were collected before the WHO 2012 VA standard was available, but were transformed into the WHO 2012 and InterVA-4 format to optimise cross-site standardisation in cause of death attribution. For a small proportion of deaths, VA interviews were not successfully completed; a few others contained inadequate information to arrive at a cause of death. InterVA-4 assigns causes of death (maximum 3) with associated likelihoods; thus, cases for which likely causes did not total to 100% were also assigned a residual indeterminate component. This served as a means of encapsulating uncertainty in cause of death at the individual level within the overall dataset, as well as accounting for 100% of every death.

**Overall dataset**

The overall public domain dataset (7) thus contains between one and four records for each death, with the sum of likelihoods for each individual being unity. Each record includes a specific cause of death, its likelihood, and its age–sex–time weighting.

It is important to be clear about the definitions of pregnancy status in relation to deaths among WRA. WHO (1) defines a PRD as ‘the death of a women while pregnant or within 42 days of termination of pregnancy, irrespective of the cause of death’. Further, a maternal death is defined as ‘the death of a woman while pregnant or within 42 days of termination of pregnancy, irrespective of the duration and site of the pregnancy, from any cause related to or aggravated by the pregnancy or its management but not from accidental or incidental causes’, which is therefore a subset of PRD. In these analyses, we do not use the concept of late maternal deaths.

In this dataset, in seven sites there were fewer than 10 PRD reported (mainly due to smaller sites, or shorter reporting periods), and these sites have been excluded from further analyses. One site, Nouna in Burkina Faso, did not record sufficient detail of pregnancy status in the VA data and therefore was also excluded. Thus, these analyses are based on reports from 14 sites, covering 14,198 deaths over 2,595,605 person-years, for which VAs were successfully completed in 91.1% of cases. Further details of the 14 sites included in the analyses here are available separately (12–25). As each HDSS covers a total population, rather than a sample, uncertainty intervals are not shown.

**Fig. 1:**
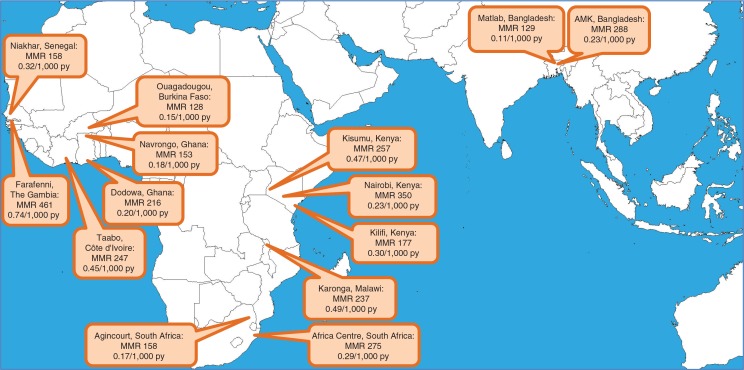
Estimated maternal mortality ratio (MMR) per 100,000 live births and maternal mortality rates per 1,000 person-years observed among women aged 15–49 at 14 INDEPTH sites.

Although the natural way to analyse these longitudinal data was in terms of pregnancy-related mortality rates per 1,000 person-years, because of the widespread use of MMR as a measure of maternal mortality, we also used data from the same populations covered by the HDSSs to generate numbers of live births, based on total person-time relating to the neonatal period. Survivors into infancy each accounted for 28 days of neonatal person-time, with smaller contributions in the case of neonatal deaths. These estimates of live births for each site were only used to produce the MMR estimates shown in Fig. 1.

Identifying maternal deaths as a subset of PRD is not entirely straightforward from VA cause of death data. Taking out the accidental causes is simple enough, but a problem remains in determining the proportion of non-obstetric, non-accidental PRD that are ‘related to or aggravated by the pregnancy or its management’, the so-called indirect maternal deaths. In clinical settings, this is a judgement that is made individually on a case-by-case basis. Anaemia as a cause of PRD is always considered to be indirect. In the absence of sufficiently detailed clinical case records to make individual judgements, but having the advantage of cause of death data for all WRA in the populations covered, we were able to estimate, for each participating site, the excess number of deaths associated with pregnancy for each specific non-obstetric, non-accidental cause, based on the proportions of PRD and non-pregnancy deaths (NPRD) from each such cause:

$$\text{excess PR}{\text{D}}_{\text{site},\text{cause}}=\text{PR}{\text{D}}_{\text{site},\text{cause}}-(\text{NPR}{\text{D}}_{\text{site},\text{cause}}\times (\text{PR}{\text{D}}_{\text{site}}/\text{PR}{\text{D}}_{\text{site}}+\text{NPR}{\text{D}}_{\text{site}}))$$

Although for some causes there may be a negative excess (corresponding to a ‘healthy-pregnancy’ effect), that is not relevant to the calculations here, because in standard clinical determination of indirect maternal deaths, no attention is given to enhanced survival from particular causes.

In this context, all of these data are secondary datasets derived from primary data collected separately by each participating site. In all cases, the primary data collection was covered by site-level ethical approvals relating to on-going health and demographic surveillance in those specific locations. No individual identity or household location data were included in the secondary data and no specific ethical approvals were required for these pooled analyses.

## Results

In a total of 14,198 WRA deaths during 2,595,605 person-years of observation, 12,939 had VA interviews successfully completed, of which 1,222 (9.4%) were pregnancy related. Of the overall person-time observed, 67.4% related to the period 2006–2012. Direct obstetric causes were involved in 472.8 (38.7%) of the 1,222 PRD, and a further 177.1 deaths (14.5%) were estimated to be due to indirect maternal causes. Thus, there were 572.1 PRD (46.8%) (so-called ‘incidental’ deaths) which were not ascribed to maternal causes. The numbers of deaths and person-years of exposure for each site are shown in Table 1.

**Table 1:** Number of deaths by site and pregnancy status, for 12,939 deaths among women aged 15–49 for whom a verbal autopsy interview was successfully completed, with person-time observed

		Pregnancy-related deaths				
		
Site	Non-pregnancy deaths	Total	Direct maternal	Indirect maternal	Incidental	Person-years observed
Bangladesh: Matlab	576	77	48.5	7.8	20.7	490,544
Bangladesh: AMK	172	43	28.1	4.4	10.5	144,278
Burkina Faso: Ouagadougou	71	13	5.7	3.3	4.1	58,795
Côte d'Ivoire: Taabo	63	15	6.5	4.9	4.6	22,867
The Gambia: Farafenni	173	90	48.5	9.8	31.7	78,447
Ghana: Navrongo	888	79	43.5	6.1	29.4	279,802
Ghana: Dodowa	447	48	25.2	2.7	20.1	140,074
Kenya: Kilifi	423	142	53.2	17.6	71.2	234,111
Kenya: Kisumu	2,801	304	73.3	44.8	185.9	252,339
Kenya: Nairobi	693	101	35.3	15.3	50.4	223,061
Malawi: Karonga	301	41	21.9	8.3	10.8	61,411
Senegal: Niakhar	120	18	10.9	4.3	2.8	48,089
South Africa: Agincourt	2,268	143	37.1	22.8	83.1	350,944
South Africa: Africa Centre	2,721	108	35.1	26.2	46.7	210,841

Figure 1 shows maternal mortality rates by site together with MMR results based on the total 649.9 maternal deaths (excluding incidental deaths) and corresponding 307,274 live births. MMRs varied between 128 and 461 per 100,000 live births, while maternal mortality rates ranged from 0.11 to 0.74 per 1,000 person-years observed among WRA.

Table 2 shows mortality rates by major cause of death categories and pregnancy status, by site. There were major variations between sites in non-pregnancy mortality rates, as reflected in other papers in this series (8, 26, 27). In particular here, there was a 50-fold range in infectious mortality rates unrelated to pregnancy. Table 3 shows mortality rates per 1,000 person-years by detailed cause of death and site, together with the overall proportion of deaths for each cause that was pregnancy related.

**Table 2:** Cause-specific mortality rates per 1,000 person-years for 12,939 deaths among women aged 15–49 years for whom a verbal autopsy interview was successfully completed, from 14 INDEPTH Network HDSS sites, by cause of death categories and pregnancy status

	Non-pregnancy mortality					Pregnancy-related mortality						All causes	
	
	Infections	Neoplasms	NCDs	External causes	Indeterminate	Infections	Neoplasms	NCDs	Obstetric causes	External causes	Indeterminate	Non-pregnancy related	Pregnancy related
Bangladesh: Matlab	0.22	0.25	0.38	0.20	0.12	0.02	0.00	0.03	0.10		0.01	1.17	0.16
Bangladesh: AMK	0.21	0.26	0.36	0.28	0.08	0.03	0.01	0.05	0.20		0.01	1.19	0.30
Burkina Faso: Ouagadougou	0.42	0.28	0.32	0.05	0.14	0.08		0.04	0.10		0.01	1.21	0.23
Côte d'Ivoire: Taabo	1.85	0.11	0.54		0.26	0.29			0.32		0.05	2.76	0.66
The Gambia: Farafenni	1.28	0.23	0.29	0.08	0.32	0.23	0.03	0.13	0.62		0.15	2.20	1.16
Ghana: Navrongo	0.96	0.73	0.47	0.33	0.69	0.02	0.01	0.03	0.16	0.01	0.06	3.18	0.29
Ghana: Dodowa	1.71	0.32	0.52	0.17	0.47	0.09		0.03	0.19		0.04	3.19	0.35
Kenya: Kilifi	1.11	0.18	0.26	0.13	0.12	0.22	0.02	0.07	0.24	0.01	0.05	1.80	0.61
Kenya: Kisumu	8.61	0.48	0.96	0.17	0.87	0.63	0.04	0.08	0.33		0.13	11.09	1.21
Kenya: Nairobi	2.20	0.10	0.28	0.24	0.29	0.17	0.00	0.07	0.17		0.05	3.11	0.46
Malawi: Karonga	3.57	0.39	0.46	0.14	0.34	0.16	0.05	0.05	0.36		0.06	4.90	0.68
Senegal: Niakhar	1.34	0.20	0.31	0.03	0.62	0.06			0.29		0.03	2.50	0.38
South Africa: Agincourt	4.33	0.39	0.47	0.36	0.91	0.17	0.00	0.05	0.12	0.01	0.06	6.46	0.41
South Africa: Africa Centre	11.03	0.50	0.50	0.45	0.42	0.18	0.02	0.05	0.18	0.00	0.07	12.90	0.50

**Table 3:** Cause-specific mortality rates per 1,000 person-years for 12,939 deaths among women aged 15–49 for whom a verbal autopsy interview was successfully completed, from 14 INDEPTH Network HDSS sites, also showing for each cause of death the proportion of pregnancy-related cases

Cause of death	Bangladesh: Matlab	Bangladesh: AMK	Burkina Faso: Ouagadougou	Côte d'Ivoire: Taabo	Ghana: Navrongo	Ghana: Dodwa	The Gambia: Farafenni	Kenya: Kilifi	Kenya: Kisumu	Kenya: Nairobi	Malawi: Karonga	Senegal: Niakhar	South Africa: Agincourt	South Africa: Africa Centre	% Pregnancy related
01.01 Sepsis (non-obstetric)	0.006	0.013		0.030	0.021				0.006	0.003			0.011		0.00
01.02 Acute resp infect, incl pneumonia	0.140	0.128	0.033	0.201	0.252	0.407	0.755	0.088	1.271	0.199	1.087	0.254	0.898	0.366	11.06
01.03 HIV/AIDS-related death	0.010		0.315	0.871	1.064	0.633	0.637	0.879	9.236	2.208	5.032	1.322	7.851	7.701	4.63
01.04 Diarrhoeal diseases	0.028		0.016	0.138	0.125	0.128	0.071	0.024	0.101	0.010	0.038	0.409	0.112	0.037	3.98
01.05 Malaria			0.034	0.587	0.057	1.471	0.854	0.045	1.611	0.044	0.615	0.412	0.252	0.054	12.47
01.07 Meningitis and encephalitis	0.017	0.022	0.063		0.231	0.160	0.385	0.047	0.350	0.219	0.193	0.184	0.076	0.394	6.63
01.09 Pulmonary tuberculosis	0.275	0.304	0.032	0.226	0.314	0.954	1.116	0.245	5.845	2.326	1.369	0.651	4.094	12.950	3.38
01.99 Other and unspecified infect dis	0.017	0.009		0.084	0.019	0.205	0.032	0.002	0.174	0.032		0.007	0.042	0.021	9.63
02.01 Oral neoplasms	0.019	0.010	0.011		0.057	0.011	0.028	0.007	0.056	0.010		0.020	0.032	0.038	4.22
02.02 Digestive neoplasms	0.231	0.259	0.096	0.035	0.932	0.510	0.406	0.064	0.346	0.029	0.355	0.131	0.333	0.207	2.05
02.03 Respiratory neoplasms	0.074	0.116	0.067	0.038	0.067	0.083	0.016	0.057	0.253	0.069		0.054	0.266	0.266	6.89
02.04 Breast neoplasms	0.076	0.040	0.046	0.037	0.310	0.078	0.114	0.008	0.082	0.018	0.086	0.185	0.206	0.136	1.01
02.06 Reproductive neoplasms	0.063	0.057	0.059		0.216	0.084	0.276	0.043	0.166	0.053	0.549	0.144	0.268	0.342	4.47
02.99 Other and unspecified neoplasms	0.041	0.070				0.023	0.014	0.025	0.136	0.021		0.014	0.060	0.047	2.91
03.01 Severe anaemia	0.024			0.043	0.005	0.021	0.016		0.038	0.007	0.102			0.020	19.30
03.02 Severe malnutrition	0.003			0.021	0.027	0.021		0.020	0.028		0.062		0.008	0.016	3.76
03.03 Diabetes mellitus	0.021	0.012	0.018	0.054	0.047	0.005		0.011	0.025	0.009	0.040		0.156	0.077	6.34
04.01 Acute cardiac disease	0.024	0.057	0.029		0.062	0.261	0.021	0.011	0.062	0.057	0.074		0.020	0.031	11.86
04.03 Sickle cell with crisis			0.007					0.004	0.010						100.00
04.02 Stroke	0.268	0.238	0.131	0.084	0.115	0.269	0.322	0.062	0.136	0.031	0.229		0.254	0.122	6.27
04.99 Other and unspecified cardiac dis	0.115	0.167	0.017	0.072	0.117	0.071	0.034	0.086	0.378	0.433	0.058		0.233	0.435	13.15
05.01 Chronic obstructive pulmonary dis	0.007	0.049			0.017			0.008	0.009			0.027	0.159	0.028	10.69
05.02 Asthma	0.043	0.016			0.008	0.071	0.006	0.033	0.060	0.020		0.066	0.372	0.058	17.57
06.01 Acute abdomen	0.194	0.162	0.144	0.162	0.451	0.487	0.408	0.047	0.751	0.104	0.467	0.434	0.178	0.172	6.55
06.02 Liver cirrhosis	0.025	0.078		0.028	0.045	0.029	0.025	0.003	0.084		0.009		0.049	0.032	15.48
07.01 Renal failure	0.018	0.013	0.006	0.033	0.030		0.027	0.003	0.064				0.008	0.081	11.54
08.01 Epilepsy	0.019	0.014		0.043	0.058	0.050	0.119	0.014	0.051	0.016	0.023	0.025	0.057	0.037	3.30
98 Other and unspecified NCD	0.042	0.049	0.008		0.017	0.014	0.055	0.029	0.406	0.023	0.042		0.083		10.26
10.06 Congenital malformation									0.008						0.00
12.01 Road traffic accident	0.018	0.033	0.016		0.309	0.220	0.116	0.046	0.062	0.116	0.078		0.292	0.284	0.62
12.02 Other transport accident	0.012														0.00
12.03 Accid fall	0.003	0.004	0.013		0.106	0.048	0.212	0.008	0.015	0.013	0.024				0.00
12.04 Accid drowning and submersion	0.024				0.021	0.034	0.129	0.016	0.058		0.026				0.00
12.05 Accid expos to smoke, fire, and flame	0.012	0.016			0.008			0.008	0.015	0.166			0.040	0.024	0.00
12.06 Contact with venomous plant/animal	0.004	0.012			0.101	0.026	0.138		0.032						0.00
12.10 Exposure to force of nature			0.017												0.00
12.07 Accid poisoning and noxious subs	0.004								0.010	0.008			0.002	0.011	0.00
12.08 Intentional self-harm	0.254	0.469			0.090	0.025		0.025	0.079	0.046	0.074	0.018	0.379	0.121	1.06
12.09 Assault	0.069	0.032			0.080	0.061		0.030	0.070	0.100	0.053	0.020	0.372	0.461	2.83
12.99 Other and unspecified external CoD									0.003						0.00
09.01 Ectopic pregnancy					0.012	0.035	0.008	0.003	0.020		0.018		0.005	0.032	100.00
09.02 Abortion-related death					0.042	0.082	0.069	0.009	0.059	0.025	0.077		0.004	0.015	100.00
09.03 Pregnancy-induced hypertension	0.080	0.069	0.017	0.082	0.036	0.054	0.229	0.060	0.074	0.027	0.053	0.067	0.048	0.117	100.00
09.04 Obstetric haemorrhage	0.073	0.223	0.073	0.122	0.156	0.226	0.693	0.127	0.251	0.095	0.315	0.346	0.167	0.077	100.00
09.05 Obstructed labour	0.006			0.010	0.010	0.003	0.013		0.004		0.025		0.013		100.00
09.06 Pregnancy-related sepsis	0.024	0.041		0.039	0.028		0.516	0.024	0.090	0.141	0.036	0.211	0.039	0.051	100.00
09.07 Anaemia of pregnancy	0.002	0.007		0.038	0.009	0.007		0.009	0.071	0.015		0.195	0.040	0.036	100.00
09.08 Ruptured uterus	0.004										0.052				100.00
09.99 Other and unspecified maternal CoD	0.012	0.060	0.006	0.031	0.061	0.042	0.484	0.005	0.088	0.047	0.119		0.038	0.027	100.00
99 Indeterminate	0.263	0.181	0.154	0.304	1.567	1.329	1.559	0.177	2.016	0.749	0.824	1.538	2.891	0.967	10.67

**Fig. 2:**
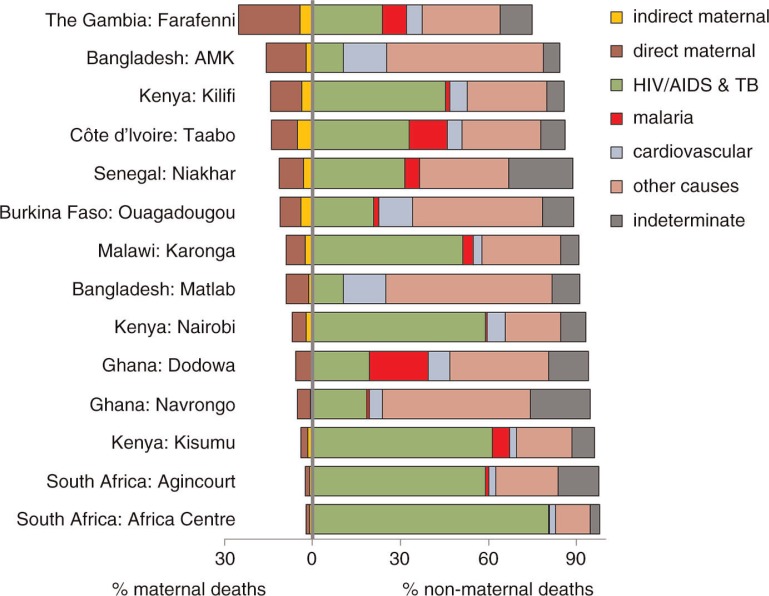
Proportions of maternal and non-maternal mortality among women aged 15–49 by cause category for 14 INDEPTH sites.

Figure 2 shows the proportions of maternal deaths (direct and indirect) for each site, along with proportions of non-maternal deaths in major cause groups. Major contributors to indirect maternal deaths were anaemia, pneumonia, malaria, and cardiovascular causes. The figure shows a substantial variation by site in the proportion of maternal deaths out of all WRA deaths (represented by the overall 100% bar for each site). Generally, sites with higher overall WRA mortality rates (Table 2), and particularly those with a substantial HIV/AIDS and tuberculosis mortality component, consequently had lower proportions of maternal deaths.

**Fig. 3:**
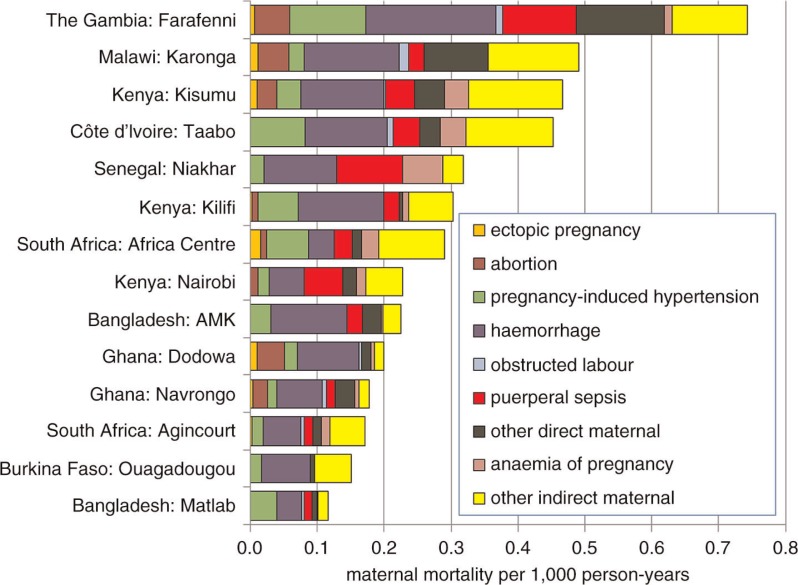
Maternal mortality rates per 1,000 person-years by WHO 2012 VA cause categories for 14 INDEPTH sites.

Figure 3 shows a detailed breakdown of maternal mortality rates by various obstetric and indirect causes. Obstetric haemorrhage was the dominant direct obstetric cause at most sites.

Although not influencing the other results presented here, it was also possible to use the same methodology to see which causes of death might be related to a ‘healthy-pregnancy’ effect, by being under-represented among the PRD. The effects were not huge, but most sites reported proportionately less cancer deaths among pregnant women; in some sites, there were less HIV/AIDS and tuberculosis deaths. Sites in Bangladesh reported lower rates of suicides among pregnant women.

## Discussion

These analyses of pregnancy-related mortality, other than being numerically large and geographically wide, were strengthened by having cause-specific mortality and pregnancy status data for all deaths of women aged 15–49 in the site populations, rather than being based on surveys of maternal deaths. Thus, even though VA is not a method that facilitates distinguishing between indirect maternal deaths and incidental pregnancy-related mortality on an individual basis, it was possible using this dataset to account for maternal deaths in several different ways, including MMR; cause-specific mortality rates; and direct, indirect, and incidental maternal deaths.

Although the natural way to analyse and present maternal mortality from a longitudinal population-based dataset of this kind is in terms of mortality rates, in Fig. 1 we also presented MMR estimates from these data in order to provide comparability with other sources of information. Although the 14 sites reporting here covered different surveillance periods, most of the data reported related to the period 2006–2012. Thus, we have compared MMR findings with those reported in UN estimates for 2010 (28). For half of the sites, the MMR point estimates in Fig. 1 lay within the range estimated for the country in 2010 by the United Nations. Other sites had MMR estimates slightly below the lower limit of the UN estimates. However, there is a lack of precision in comparing specific HDSS areas with national estimates.

The concepts of PRD that are ‘indirectly’ due to pregnancy or ‘incidental’ to pregnancy are somewhat fraught, and have become all the more difficult to interpret in populations with high HIV/AIDS mortality burdens. Because conceiving and successfully maintaining a pregnancy tend to require being reasonably healthy in the first place, the resulting selection effect means that pregnancy can appear to reduce women's mortality from many causes (29). Certainly care has to be taken in interpreting population proportions of maternal mortality depending on other mortality pressures such as HIV/AIDS and malaria, as is evident from Fig. 2. Although the two South African sites (Agincourt and Africa Centre) in the figure had very low proportions of maternal deaths compared with overall WRA mortality, their MMRs were by no means low; but the massive burdens of HIV/AIDS mortality in the WRA populations in those locations minimised the maternal proportions. We believe that the approach we have taken for estimating indirect maternal deaths on the basis of specific cause proportions among pregnancy-related and non-pregnancy deaths is effective where VA data are available for all WRA deaths in a population. It offers a significant advantage over the standard DHS methods, which can only measure pregnancy-related mortality, rather than maternal mortality (3).

There has also been considerable discussion, particularly in relation to arriving at realistic global estimates of maternal mortality, as to the role of HIV infection in PRD. Although it is well established that HIV-positive women are less likely to become pregnant, and that they are substantially more likely to die from numerous causes irrespective of pregnancy (30), it has been a challenge to ascertain the mortality risks to HIV-positive women who do become pregnant, in terms of possible interactions between HIV positivity and pregnancy (31). Our results, shown in Fig. 2, suggest that HIV/AIDS does not constitute a major proportion of indirect maternal mortality, even in settings with high HIV/AIDS mortality burdens.

Apart from the obvious limitations of VA in any context, its use in relation to maternal mortality depends crucially on ascertaining pregnancy status reliably in the VA interview. Depending on how the VA interview is carried out, and who the available respondent is, there may be difficulties around capturing pregnancy status, particularly in relation to early or undisclosed pregnancies. Most sites, however, reported some cases of ectopic pregnancy and/or abortion-related deaths, which may indirectly be indicators of data reliability in relation to pregnancy. The proportions of direct maternal, indirect maternal, and incidental deaths making up total PRD here also suggests that many deaths not self-evidently connected with pregnancy were indeed identified as being pregnancy related in the VA interviews. Although it might be argued that prospective pregnancy registration could improve detection of pregnancies, and this is done in some INDEPTH sites, it is a hugely resource-intensive undertaking, probably only applicable in research settings. On the contrary, analysing total WRA cause of death data, which could in principle come from VA used in the context of civil registration of deaths, provides a more direct method for analysing and documenting maternal mortality (32).

## Conclusions

Although there are many potential difficulties in measuring maternal mortality at the population level, our findings here are generally plausible and in line with other estimates. They confirm the continuing unacceptably high levels of mortality in women in conjunction with giving birth across Africa and in parts of Asia. Measuring these high rates by recording the individual tragedies involved is not the solution to the problem, but understanding the details of what is happening at the population level is a pre-requisite to implementing and evaluating solutions.
